# Humoral response to SARS‐CoV‐2 and seasonal coronaviruses in COVID‐19 patients

**DOI:** 10.1002/jmv.27427

**Published:** 2021-11-08

**Authors:** Ortwin Adams, Marcel Andrée, Denise Rabl, Philipp N. Ostermann, Heiner Schaal, Erik Lehnert, Stefanie Ackerstaff, Lisa Müller, Johannes C. Fischer

**Affiliations:** ^1^ Institute for Virology, Medical Faculty Heinrich‐Heine‐University of Duesseldorf Düsseldorf Germany; ^2^ Institute for Transplantation Diagnostics and Cell Therapeutics, Medical Faculty Heinrich‐Heine‐University of Duesseldorf Düsseldorf Germany

**Keywords:** humoral immune response, SARS‐CoV‐2, seasonal coronaviruses

## Abstract

We used enzyme‐linked immunoassay methods to measure the prevalence and the levels of antibody responses to the nucleocapsid (N) protein of severe acute respiratory syndrome coronavirus 2 (SARS‐CoV‐2) and four seasonal human coronaviruses (HCoV‐OC43, HCoV‐HKU1, HCoV 229E, and HCoV‐NL63) in a cohort of 115 convalescent plasma donors infected with SARS‐CoV‐2 (1–61 days after symptom onset) compared to antibody levels in 114 individuals with no evidence of a recent infection with SARS‐CoV‐2. In the humoral response to the four seasonal coronaviruses, only HCoV‐HKU1‐ and HCoV‐229E‐assays showed slightly elevated antibody levels in the COVID group compared to the control group. While in the COVID‐group the levels of SARS‐CoV‐2 antibodies correlated significantly with disease severity, no association was found in the levels of antibodies against the seasonal coronaviruses. The most striking result in both groups was that the levels of antibodies against all tested coronaviruses, including the new SARS‐CoV‐2 showed a highly significant correlation with each other. There seems to be an individual predisposition to a weaker or stronger humoral immune response against all known seasonal human coronaviruses including the new SARS‐CoV‐2, which could lead to a definition of low and high responders against human coronaviruses with potential impact on the assessment of postinfection antibody levels and protection.

## 1 BACKGROUND

At the end of December 2019, the first cases of severe pneumonia with an unknown cause were reported in Wuhan, China.^1^, ^2^ The new virus is very similar to the beta‐coronavirus SARS‐coronavirus and was, therefore, named severe acute respiratory syndrome coronavirus 2 (SARS‐CoV‐2). SARS‐CoV‐2 is the cause of the current COVID‐19 pandemic.^3^ In addition to SARS‐CoV‐2, we know of six other human coronaviruses today. Among them are four seasonal human coronaviruses, that widely circulate in the human population and are responsible for 10%–30% of mild self‐limiting upper respiratory tract infections.^4^

A protective effect of previous infections with seasonal human coronaviruses against infection with SARS‐CoV‐2 has been described as well as an association between previous infections with seasonal human coronaviruses and less severe COVID‐19 disease.^5^, ^6^ However, in first case the studies are based only on presumed infections due to the presence of clinical symptoms. In the latter case PCR proven infections with a seasonal human coronavirus are associated with less severe disease manifestations from SARS‐CoV‐2 infection.

To more clearly determine the effect of previous infections with human seasonal coronaviruses on the severity of disease and the humoral immune response to infections with SARS‐CoV‐2 we used enzyme‐linked immunosorbent assay (ELISA) based immunoassays for the detection of antibodies against five different human coronaviruses and tested sera from SARS‐CoV‐2 convalescent plasma donors and a SARS‐CoV‐2 unexposed control group.

## 2 PATIENTS AND METHODS

### 2.1 Study population

Characteristics of the study population were summarized in Table 1. The study was approved by the local ethics committee al Heinrich‐Heine University Duesseldorf (study no. 2020‐1148). Informed consent was obtained from all of the SARS‐CoV‐2 convalescent plasma donors (*N* = 115) before blood sampling. The control sera (*N* = 114) derived from routine presurgery serology testing excluding patients with known underlying chronic diseases or immunosuppressive therapy. The basic demographics were similar for both groups (Table 1). Severity of disease was classified as 0 for patients without any symptoms (*n* = 5, corresponding to WHO COVID‐19 ordinal scale °1), as 1 in patients with only mild symptoms and no relevant restriction of activities (*n* = 28, °1), 2 for patient with restriction of activities (corresponding to WHO°2a [*n* = 44]), and 3 for patients with more severe symptoms (corresponding to WHO°2b (*n* = 16] and borderline WHO°3 [*n* = 2]). Classification information was not available for 20 patients. None of the patients was hospitalized. All blood samples of the study group and control group were collected between March 2020 and July 2020 and stored at −20°C. Additionally, for 10 post‐COVID plasma donors it was possible to obtain sequential sera for up to 6 months after onset of symptoms.

**Table 1:** Characteristics of study populations and blood samples

Characteristics	Plasma‐donors	Day after first symptoms^a^	Control‐group	*p*
Total *N* (%)	115 (100)		114 (100)	
Gender				
Male *N* (%)	60 (52)		67 (59)	1.000
Female *N* (%)	55 (48)		47 (41)	1.000
Mean years (CI)	44.5 (42.0–47.1)		46.6 (44.1–49.2)	0.252
Median years (min–max)	47.9 (19.9–77.1)		50.2 (20.3–77.9)	
Severity of disease (score) *N* (%)				
0	5 (4)	26.3 (−1.1–53.8)	n.a.	
1	28 (24)	47.0 (37.2–56.8)	n.a.	
2	44 (38)	53.9 (48.3–59.5)	n.a.	
3	18 (16)	51.0 (41.3–60.6)	n.a.	
unclassified	20 (17)			
Date of sample	March–July 2020		March–July 2020	

Abbreviation: CI, confidence interval.

^a^ The day after the onset of the disease when the blood sample was taken.

### 2.2 Enzyme‐linked immunosorbent assay

The detailed protocol for the anticoronavirus‐N‐protein GST capture ELISA was originally developed and recently published for the detection of antibodies against human polyoma virus JC and BK polyomaviruses.^7^, ^8^ Briefly, affinity‐purified SARS‐CoV‐2, HCoV‐HKU1, HCoV‐OC43, HCoV‐229E, and HCoV‐NL63‐Nucleocapsid (N) expressed as GST fusion proteins in *Escherichia coli* BL21 cells in situ on gluthation casein‐coated ELISA plates were used as antigens. RNA eluates from patient samples with PCR‐proven coronavirus‐infections of the University Hospital Düsseldorf were used for cloning the N genes with the exception of the N‐gene of HCoV‐HKU1, which was produced synthetically. To increase specificity, we chose a protocol in which cross‐reactive antibodies are bound in a preincubation step with serum antibodies and a soluble heterologous coronavirus nucleocapsid protein. So, soluble SARS‐CoV‐2‐N and HCoV‐OC43‐N bacterially expressed as fusion protein with N‐terminal maltose‐binding protein were generated for the pre‐adsorption of all sera before the detection of the antibodies. For the SARS‐CoV‐2 assay, the sera were preadsorbed with soluble HCoV‐OC43 N protein and for other assays with soluble SARS‐CoV‐2 N protein, respectively. Antigen‐coated ELISA plates were incubated for 1 h with preadsorbed sera at 1:100 dilutions, and a polyclonal anti‐human IgG peroxidase antibody (Sigma‐Aldrich) and tetramethylbenzidine (BD Bioscience) were used for detection. The optical density (OD) was measured at 450 nm. The antibody reactivity were measured in arbitrary units (AU) and the dynamic range of the ELISAs were determined by serial dilution of human immunoglobulin (Ig, Privigen®, CSL Behring GmbH) for the detection of seasonal coronavirus‐antibodies. OD values of the samples were compared to a standard curve, using curve‐fitting of point‐to‐point calculation. Since no clear threshold value could be defined for the seasonal coronaviruses that could distinguish a seropositive from a seronegative status, all AU/ml >0 were considered for statistical evaluation. For the SARS‐CoV‐2 antibody assay sera from 5 seropositve convalescent patients as determined by a commercial SARS‐CoV‐2 antibody assay were pooled and diluted. An OD_450_ of 2.0 was arbitrarily defined as 1000 AU. Additionally, sera from convalescent COVID patients were also tested by the Euroimmun‐S‐Protein antibody assay (Euroimmun; Lubeck, Germany #EI 2606‐9601 G). The antibody detection and reactivity against Measles virus were measured by a fully automated commercial ELISA (REF EI 2610‐9601‐1 G, EUROIMMUN, Luebeck, Germany) as recommended by the manufacturer.

### 2.3 SARS‐CoV‐2 neutralization antibodies

To determine the SARS‐CoV‐2 neutralization activity of the plasma samples, a serial endpoint neutralization test for SARS‐CoV‐2 was developed based on our prior work.^9^ Serial dilutions of heat‐inactivated (56°C, 30 min) serum samples in duplicates were preincubated in cell‐free plates with 100 TCID_50_ units of SARS‐CoV‐2 for 1 h at 37°C. After preincubation, 100 µl of cell suspension containing 7 × 10^4^/ml Vero cells (ATTC‐CCL‐81) were added. Plates were incubated at 37°C, 5% CO_2_ for 4 days before microscopic inspection for virus induced cytopathic effect.

### 2.4 Statistical analysis

The data were analyzed using SPSS Statistics 25 (IBM^©^) and GraphPad Prism 5.01 (GraphPad Software). Categorical data were studied using Fisher's exact test or Pearson's chi square test, depending on the sample size. The 95% confidence interval (95% CI) for proportions was calculated using the modified Wald method. Quantitative data were analyzed by the nonparametric Mann–Whitney *U* test for two groups and by the Kruskal–Wallis test for more than two groups. Correlation coefficients (*r*2) were determined to calculate the quality of the fit of the quantitative results from the different ELISA assays. All of the tests were two tailed, and *p* < 0.05 was considered to be statistically significant.

## 3 RESULTS

### 3.1 Antibody levels in convalescence individuals with SARS‐CoV‐2 infection and control group

**Figure 1:**
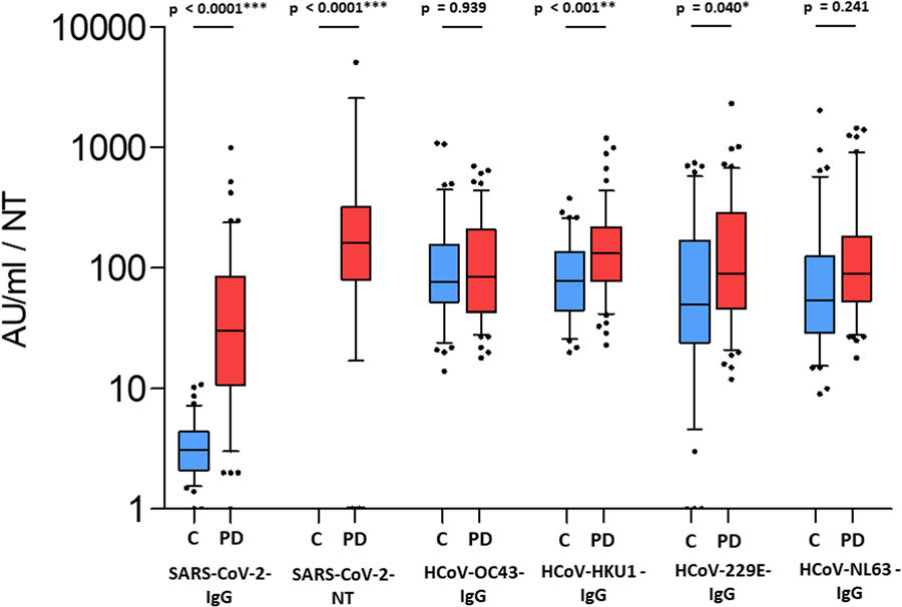
Antibody levels for SARS‐CoV‐2 and seasonal coronaviruses in the SARS‐CoV‐2 and the control group. Box‐whisker plots showing antibody levels of SARS‐CoV‐2 and seasonal coronavirus antibodies. Boxes span the interquartile range; the line within each box denotes the median and whiskers indicate the 5 and 95 percentile values. Outliers are indicated by black asteriks. Values are given in arbitrary units (AU) for the control group (C) (blue boxes) and the plasma donors (PD) of the COVID group (red boxes). *p calculated by *t* test. SARS‐CoV‐2, severe acute respiratory syndrome coronavirus 2

For validation of the SARS‐CoV‐2 GST capture ELISA the antibody levels of 111 out of 115 sera from the COVID‐group were compared to antibody levels determined in the Euroimmun anti‐Spike‐protein assay. The remaining four samples could not be analyzed due to lack of sample volume. A highly significant correlation between the assays was found (*R* = 0.572, *p* < 0.001). There were 18 out of 111 (16.2%) seronegative individuals as determined in the anti‐S‐protein assay. In the GST capture ELISA mean antibody level of the 18 sera was 12 AU/ml (2–31 AU/ml) (data not shown). The mean antibody level of the COVID group was 71.5 AU/ml (CI: 49.6–93.4 AU/ml). In contrast to the COVID‐group, only low reactivity against SARS‐CoV‐2 could be detected in the GST‐capture ELISA (mean 3.8, CI: 3.3–4.3, *p* < 0.0001) in the control group. The highest difference between the COVID and the control group could be found between antibody levels measured in the neutralisation test with a mean titer of 1: 495 (CI: 287–703) in the COVID group and no individual with detectable N‐titer at the highest serum concentration tested in the control group (*p* < 0.0001) (Figure 1). Only a tendency to slightly higher amounts of antibodies to seasonal coronaviruses were detected in the COVID as compared to the control group (Figure 1). For HCoV‐HKU1, significantly higher amounts of antibodies were found in the COVID group (173 AU/ml [CI: 142–205 AU/ml] vs. 105 AU/ml [CI: 84–126 AU/ml], *p* = 0.001) (Figure 1) in the control group. The same was true for the comparison of the two groups with HCoV‐229E (201 AU/ml [CI: 150–253 AU/ml] in the COVID group vs. 136 AU/ml [CI: 102–171 AU/ml] in the control group, *p* = 0.004). For the two other seasonal coronaviruses, HCoV‐OC43 and HCoV‐NL63, respectively no significant differences in antibody levels were found between the two groups (Figure 1). Gender showed no significant influence on antibody levels. In both groups, anitbodies against seasonal coronaviruses were slightly higher in males than in females (mean difference: 27.5 AU/ml, range from 3.3 to 57.0 AU/ml), but the difference was not significant. Likewise, age had no effect on the level of antibodies to seasonal coronaviruses with the exception of HCoV‐HKU1 antibodies, which showed a significant increase with age (*p* = 0.041), but only in the COVID group (data not shown).

### 3.2 Antibody levels depending on the severity of the disease

**Figure 2:**
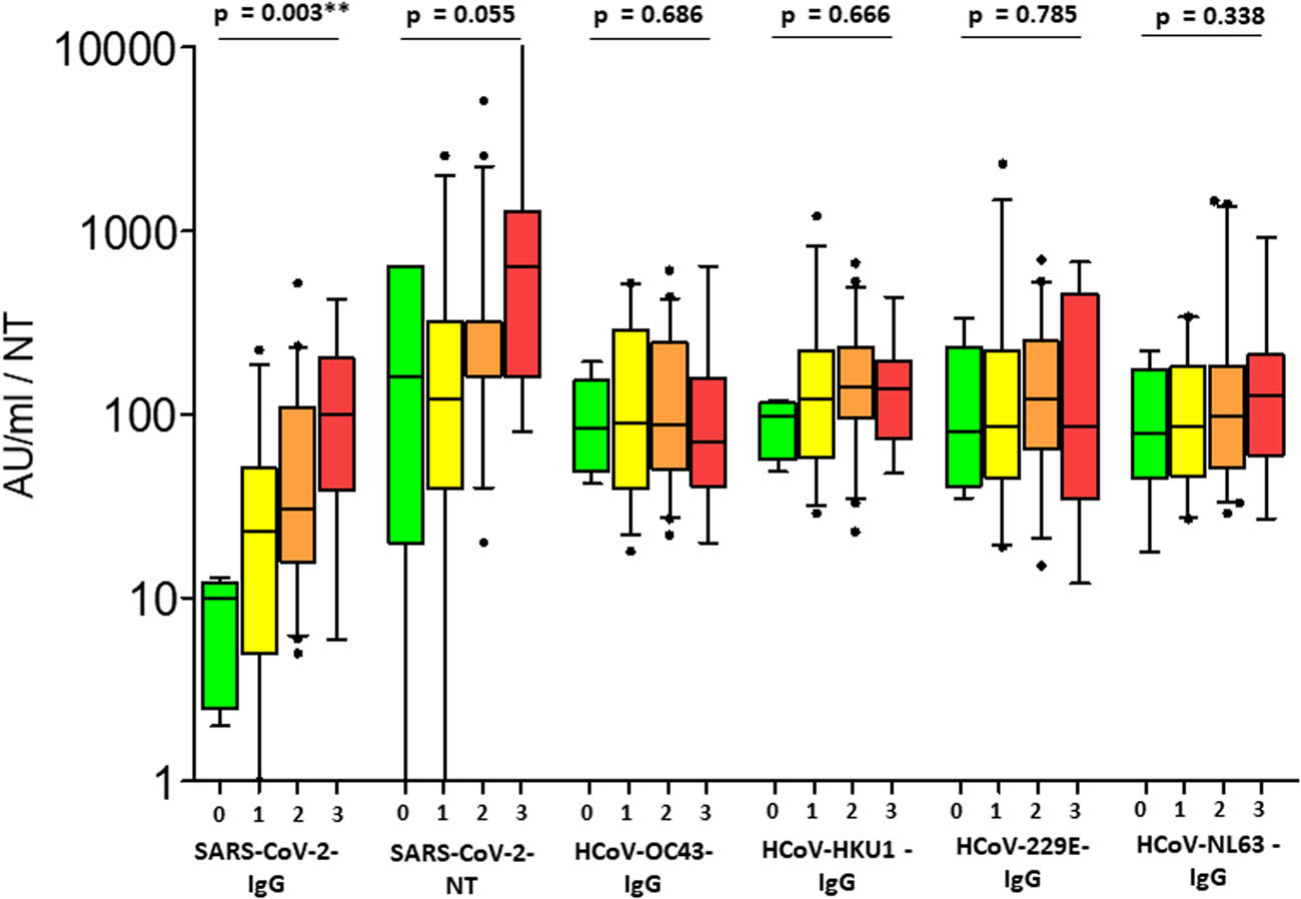
Antibody levels in reconvalescence individuals with SARS‐CoV‐2 depending on the severity of the disease. Box‐whisker plots showing antibody levels of SARS‐CoV‐2 and seasonal coronavirus antibodies. Boxes span the interquartile range; the line within each box denotes the median and whiskers indicate the 5 and 95 percentile values. Outliers are indicated by black asterisks. For each antibody species, results were grouped by the severity of disease from 0 (asymptomatic) to 3 (severe disease) as depicted at the bottom of the graph. *p calculated by one way ANOVA analysis test. ANOVA, analysis of variance;

There was a clear correlation in the COVID group between the severity of the disease and the level of SARS‐CoV‐2 antibodies as measured in the GST capture ELISA (*p* = 0.003). The small group of asymptomatic infected individuals (*N* = 5) had an average SARS‐CoV‐2 antibody level of 7.8 AU/ml (range: 2–13 AU/ml), the group with Grade 1 (*N* = 28) had a level of 40.1 AU/ml (0–224 AU/ml), the group with severity score 2 (*N* = 44) of 70.7 AU/ml (5–520 AU/ml) and finally the group with severity score 3 (*N* = 17) of 131 AU/ml (6–421 AU/ml) (Figure 2). Neutralizing antibodies showed a clear trend towards higher antibody levels with increasing symptoms, but only reached the level of weak significance (*p* = 0.055) (Figure 2). In contrast, the antibody levels against the four seasonal coronaviruses showed no significant difference or even a trend. The median antibody level was around 100 AU/ml in all cases (Figure 2). The mean age of the Grade 1–3 groups showed an increase from 39.12 to 48.67 years, but the differences were not statistically significant (data not shown).

### 3.3 Correlation analysis of all coronavirus antibodies in both groups

The most striking result of this study was a high correlation of antibody titers between all coronavirus types (Tables 2 and 3). In the group of COVID patients, highly significant correlations (*p* < 0.0001) were found between the level of SARS‐CoV‐2 antibodies and seasonal coronaviruses (Table 2) with HCoV‐HKU1 having the highest correlation coefficient (*R* = 0.514). However, the Pearson correlation coefficients show overall rather moderate values between 0.3 and 0.4, slightly higher against HCoV‐HKU1 with a value of 0.514. There were also highly significant correlations between all other antibody levels of seasonal coronaviruses, highly significant correlations were also found with the highest Pearson correlation coefficient found between HCoV‐OC43 and HCoV‐HKU1 (*R* = 0.649), followed by the correlation between HCoV‐HKU1 and HCoV‐229E (*R* = 0.624). An equally clear correlation was also found in the relationship between all the four seasonal coronaviruses within the control group (Table 3). The highest correlation coefficient in the control group was found in the relationship between HCoV‐OC43 and HCoV‐HKU1 (*R* = 0.808).

**Table 2:** Correlation analysis of the different human coronaviruses (Pearson correlation coefficient [*p* value]) in the COVID‐group

	SARS‐CoV‐2‐IgG	SARS‐CoV‐2‐NT	HCoV‐OC43‐IgG	HCoV‐HKU1‐IgG	HCoV‐229E‐IgG	HCoV‐NL63‐IgG	Measles‐IgG
SARS‐CoV‐2‐IgG	1.000	0.240 (***0.009***)	0.327 (***0.000***)	0.514 (***0.000***)	0.375 (***0.000***)	0.399 (***0.000***)	0.040 (0.686)
SARS‐CoV‐2‐NT		1.000	0.144 (0.122)	0.096 (0.302)	0.099 (0.289)	0.103 (0.268)	0.285 (***0.003***)
HCoV‐OC43‐IgG			1.000	0.649 (***0.000***)	0.434 (***0.000***)	0.325 (***0.000***)	−0.060 (0.544)
HCoV‐HKU1‐IgG				1.000	0.624 (***0.000***)	0.382 (***0.000***)	0.007 (0.942)
HCoV‐229E‐IgG					1.000	0.430 (***0.000***)	−0.068 (0.493)
HCoV‐NL63‐IgG						1.000	−0.045 (0.650)

*Note*: A *p* value of <0.05 is assumed to be significant (shown in bold/italic).

Abbreviations: IgG, Immunoglobulin G; SARS‐CoV‐2, severe acute respiratory syndrome coronavirus 2.

**Table 3:** Correlation analysis of the different human coronaviruses (Pearson correlation coeffizient [*p* value]) in the control group

	HCoV‐OC43‐IgG	HCoV‐HKU1‐IgG	HCoV‐229E‐IgG	HCoV‐NL63‐IgG	Measles‐IgG
HCoV‐OC43‐IgG	1.000	0.808 (***0.000***)	0.502 (***0.000***)	0.220 (***0.019***)	0.009 (0.927)
HCoV‐HKU1‐IgG		1.000	0.398 (***0.000***)	0.249 (***0.000***)	−0.118 (0.231)
HCoV‐229E‐IgG			1.000	0.423 (***0.000***)	0.027 (0.781)
HCoV‐NL63‐IgG				1.000	−0.105 (0.288)
Measles‐IgG					1.000

*Note*: A *p* value of <0.05 is assumed to be significant (shown in bold/italic).

Abbreviation: IgG, Immunoglobulin G.

Sera from both groups were tested for the presence of antibodies to the measles virus. As expected, there was a high seroprevalence against measles (104 out of 115, 90.4% in the COVID‐group and 105 out of 114, 92.1% in the control group). In both groups, no correlation was found between antibody levels against SARS‐CoV‐2 or seasonal coronaviruses and antibodies against measles virus with the exception of SARS‐CoV‐2 antibodies in the neutralization assay (*R* = 0.285, *p* = 0.003) in the first group (Tables 2 and 3).

### 3.4 Kinetics of antibody development against SARS‐CoV‐2 and seasonal coronaviruses over time

**Figure 3:**
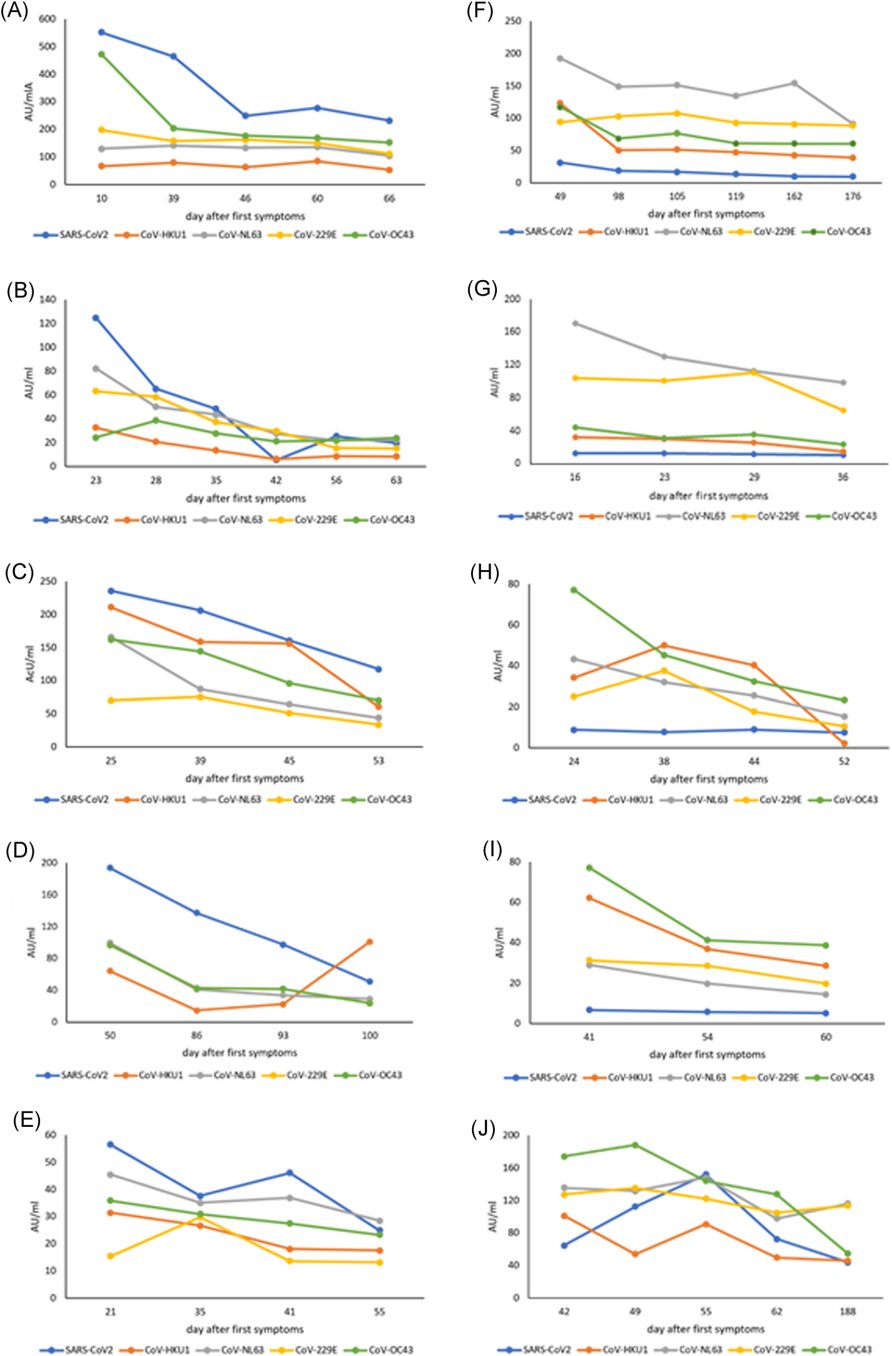
Time course of antibody levels in AU/ml for SARS‐CoV‐2 and seasonal coronaviruses in ten SARS‐CoV‐2 infected individuals. Each panel represents one of these patients (A–J). Patients A–E are SARS‐CoV‐2 high responders, Patients F–I SARS‐CoV‐2 low responders, patient J showed a delayed SARS‐CoV‐2 antibody increase (for details, see text). Each point represents a measurement of antibodies in AU/ml at a specific time after the onset of symptoms (day after first symptoms). Colors denote the different antigens that were tested as shown in the bottom line of each panel. SARS‐CoV‐2, severe acute respiratory syndrome coronavirus 2

The sequential serum samples collected from 10 plasma donors up to 6 months postonset of symptoms (range: 36–188 days) allowed for the evaluation of the kinetics and longevity of the antibody response in greater detail (Figure 3). There were significant differences in the antibody levels among the 10 individuals in the timepoints of first and last serum. The first serum was drawn at a mean of 24.5 days (range: 10–50 days) after onset of symptoms, the last serum at 61.5 days (range: 36–188 days) after onset. There was a significant decline of antibody levels against SARS‐CoV‐2 from a median of 61 AU/ml (interquartile range [IQR]: 12–205) in the first sample to 22.5 AU (IQR: 9.5–67.5) in the last sample (*p* = 0.03). This decrease was significantly greater in individuals with primarily measurable antibody responses (Figure 3A–E). Four individuals showed no specific humoral response to SARS‐CoV‐2 over the entire observation period (Figure 3F–I), one individual had an increase from 65 AU/ml to 155 AU/ml by Day 55 and then dropped again to 44 AU/ml (Figure 3J). Consistent with the results above, patients A–E (high SARS‐CoV‐2 responders, *n* = 5) showed a mean severity score of 2.2 (CI: 1.16–3.24) and a mean age of 54.7 (CI: 44.6–64.82). Patients F–I (low SARS‐CoV‐2 responders, *n* = 4) had a lower severity score (1.0, CI: −0.3–2.3) and were younger (mean age 30.65, CI: 20.2–41.1) (data not shown).

Antibody levels against the seasonal coronaviruses also showed significant decreases: for HCoV‐HKU1 from a median of 63.5 AU/ml (IQR: 33–111) to 34.0 AU/ml (IQR: 13.5–101) (*p* = 0.03), for HCoV‐OC43 from a median of 87 AU/ml (IQR: 42.8–165) to 31.5 AU/ml (IQR: 23.8–63.3) (*p* = 0.01), HCoV‐229E from a median of 82 AU/ml (IQR: 29.5–110.5) to 29 AU/ml (IQR: 14.5–114) (*p* = 0.006), and HCoV‐NL63 from a median of 115.5 AU/ml (IQR: 45.3–167) to 36.5 AU/ml (IQR: 21–116) (*p* = 0.002) (Figure 3). This drop in antibody levels was seen in both, the group of initially elevated SARS‐CoV‐2 antibodies titers (Figure 3A–E) and the group of low SARS‐CoV‐2 responders (Figure 3F–I) during the observation period.

## 4 DISCUSSION

We developed a specific two‐step capture ELISA protocol to detect antibodies against the nucleocapsid protein of the four seasonal human coronavirus and SARS‐CoV‐2 based on a technique we previously published for human polyomaviruses.^7^, ^8^ As previously shown by others, we also found a correlation between the level of antibodies to SARS‐CoV‐2 and the severity of the disease.^10^, ^11^, ^12^, ^13^ In contrast to this relationship, we found no correlation between the severity and the level of antibodies to seasonal coronaviruses. It is possible that in some viral infections, non‐neutralizing antibodies may enhance infection and not be protective against reinfection. Antibody‐dependent enhancement was discussed earlier with SARS‐CoV pathogenicity, was shown with other virus infections, e.g., Dengue virus serotypes 1–4 and is also discussed with SARS‐CoV‐2 infections.^14^, ^15^, ^16^, ^17^ As a caveat, it is noteworthy that we have shown this lack of correlation only in antibodies to the N protein and not in antibodies to other SARS‐CoV‐2 proteins. However, on the basis of the available data, we have no evidence that previous infections with seasonal coronaviruses have an impact on the severity of SARS‐CoV‐2 disease.

In the 10 donors with consecutive sera in most cases increased antibody levels against seasonal coronaviruses were found in the initial serum which decreased in the subsequent follow up. We interpreted this finding as a cross stimulation of B cells (Figure 3). This was seen in several cases with antibodies against more than one coronavirus and was also found in individuals with no or only a low level of SARS‐CoV‐2 antibodies (Figure 3F–I), thus, it is unlikely to be caused by pure cross‐reactivity of antibodies. The rapid decrease in SARS‐CoV and SARS‐CoV‐2 antibodies was described in a large number of papers, but in the present work, we were additionally able to show for the first time that this decrease also affects antibodies against seasonal coronaviruses that had initially been increased.^13^, ^18^, ^19^, ^20^, ^21^, ^22^ We cannot say from our data whether these cross‐stimulated antibodies are also functionally active, i.e., whether they can take over a protective function for the host. Furthermore, we have no information whether this cross‐stimulation of antibodies also occurs against epitopes of other viral proteins such as the spike protein of the SARS‐CoV‐2 virus.

The most striking finding of the present study was the highly significant correlation of antibody levels among all coronaviruses including in the COVID group antibodies against SARS‐CoV‐2. The correlation with the latter is somewhat lower what could be due to the fact that the COVID patients were still in the postinfectious period where other factors such as the time of blood collection and the severity of the disease are influencing the level of antibodies. It can be speculated that the SARS‐CoV‐2 antibody levels of these patients will adjust to seasonal coronavirus antibody levels in a later postinfectious period. As already shown in other studies, in the post‐COVID sera we found large individual differences in the level of antibodies with up to 10%–20% of SARS‐CoV‐2 recovered individuals who do not possess neutralizing antibodies and also did not form antibodies against other viral proteins.^13^, ^20^, ^23^, ^24^ Furthermore, it is not yet clear whether the decline of antibody levels after infection also translates to lost protection against re‐infection with SARS‐CoV‐2. Based on our finding that individuals with no or only low immune response against SARS‐CoV‐2 also show low antibody levels against seasonal coronaviruses, we argue that there is an individually adapted humoral immune response against the entire family of human coronaviruses. Accordingly, one could define high‐ and low coronavirus‐responders, whereas the latter are obviously not protected worse, but only need lower levels of antibodies to control the virus. This observation may be important for follow‐up studies with respect to assessment of SARS‐CoV‐2 vaccine‐induced protection by the humoral immune system. However, this does not seem to reflect a general characteristic of the host to noncoronavirus viral infections, because we found no correlation with the level against the measles virus. Measles antibodies were chosen due to the greater than 90% seroprevalence in adults.

Another interpretation of our data leads to the term of “trained immunity”: There is evidence, that influenza vaccine can induce trained immunity responses against SARS‐CoV‐2, which may result in relative protection against COVID‐19.^25^ Accordingly, our results could be interpreted as trained immunity induced by previous infections with seasonal coronaviruses. It could therefore make sense to determine the pre‐existing immunity to seasonal coronaviruses before vaccinating against SARS‐CoV‐2, to assess, whether a low response to a vaccine simply reflects a general constitution of the patient as a coronavirus low‐responder.

## CONFLICT OF INTERESTS

The authors declare that there are no conflicts of interests.

## AUTHOR CONTRIBUTIONS

*Conceptualization*: Ortwin Adams, Marcel Andrée, Lisa Müller, and Johannes C. Fischer. *Laboratory analysis*: Denise Rabl. *Writing—original draught preparation*: Ortwin Adams, Marcel Andrée, Lisa Müller, Philipp N. Ostermann, and Heiner Schaal. *Writing—review and editing*: Ortwin Adams, Marcel Andrée, Lisa Müller, Philipp N. Ostermann, Erik Lehnert, Stefanie Ackerstaff, Heiner Schaal. *Sample collection*: Erik Lehnert, Stefanie Ackerstaff, and Denise Rabl. *Development and implementation of serological testing*: Ortwin Adams, Marcel Andrée, Lisa Müller, Denise Rabl. *Analyzing clinical data*: Erik Lehnert,Stefanie Ackerstaff, Johannes C. Fischer, all authors read and critically revised the manuscript.

## Data Availability

The data are not publicly available due to privacy and ethical restrictions.

## References

[1] Zhu S , Chen J , Zhao J , et al. Genomic insights on the contribution of balancing selection and local adaptation to the long‐term survival of a widespread living fossil tree, Cercidiphyllum japonicum. New Phytol. 2020;228:1674‐1689. 32643803 10.1111/nph.16798

[2] Zhu N , Zhang D , Wang W , et al. A novel coronavirus from patients with pneumonia in China, 2019. N Engl J Med. 2020;382(8):727‐733. 31978945 10.1056/NEJMoa2001017 PMC7092803

[3] Hoehl S , Ciesek S . [The virology of SARS‐CoV‐2]. Pneumologe (Berl). 2020:1‐4. doi:10.1007/s10405-020-00358-x 10.1007/s10405-020-00358-x PMC7585742 33132795

[4] de Wilde AH , Snijder EJ , Kikkert M , van Hemert MJ . Host factors in coronavirus replication. Curr Top Microbiol Immunol. 2018;419:1‐42. 28643204 10.1007/82_2017_25 PMC7119980

[5] Aran D , Beachler DC , Lanes S , Overhage JM . Prior presumed coronavirus infection reduces COVID‐19 risk: a cohort study. J Infect. 2020;81:923‐930. 33127456 10.1016/j.jinf.2020.10.023 PMC7590640

[6] Sagar M , Reifler K , Rossi M , et al. Recent endemic coronavirus infection is associated with less severe COVID‐19. J Clin Invest. 2021;131(1):e143380. 10.1172/JCI143380 PMC7773342 32997649

[7] Warnke C , Dehmel T , Posevitz‐Fejfár A , et al. Anti‐JC‐virus antibody prevalence in a German MS cohort. MultScler. 2012;18(7):1054‐1055. 10.1177/1352458511429955 22740609

[8] Warnke C , Pawlita M , Dehmel T , et al. An assay to quantify species‐specific anti‐JC virus antibody levels in MS patients. MultScler. 2013;19:1137‐1144. 10.1177/1352458513475489 23388163

[9] Babor F , Grund S , Siepermann M , et al. Epidemiology and clinical characteristics of pandemic (H1N1) 2009 influenza infection in pediatric hemato‐oncology and hematopoietic stem cell transplantation patients. Transpl Infect Dis. 2012;14(6):589‐594. 23013490 10.1111/tid.12013

[10] Choe PG , Kang CK , Suh HJ , et al. Antibody responses to SARS‐CoV‐2 at 8 Weeks Postinfection in Asymptomatic Patients. Emerging Infect Dis. 2020;26(10):2484‐2487. 10.3201/eid2610.202211 PMC7510710 32579877

[11] Ko JH , Joo EJ , Park SJ , et al. Neutralizing antibody production in asymptomatic and mild COVID‐19 patients, in comparison with pneumonic COVID‐19 patients. J Clin Med. 2020;9(7):131. 10.3390/jcm9072268 PMC7408950 32708872

[12] Long QX , Liu BZ , Deng HJ , et al. Antibody responses to SARS‐CoV‐2 in patients with COVID‐19. Nat Med. 2020;26(6):845‐848. 32350462 10.1038/s41591-020-0897-1

[13] Ripperger TJ , Uhrlaub JL , Watanabe M , et al. Orthogonal SARS‐CoV‐2 serological assays enable surveillance of low‐prevalence communities and reveal durable humoral immunity. Immunity. 2020;53(5):925–933. 33129373 10.1016/j.immuni.2020.10.004 PMC7554472

[14] Yip MS , Leung HL , Li PH , et al. Antibody‐dependent enhancement of SARS coronavirus infection and its role in the pathogenesis of SARS. Hong Kong Med J. 2016;22(3 Suppl 4):25‐31. 27390007

[15] Halstead SB . Dengue antibody‐dependent enhancement: knowns and unknowns. Microbiol Spectr. 2014;2(6). 10.1128/microbiolspec.AID-0022-2014 26104444

[16] Halstead SB . Dengue hemorrhagic fever: two infections and antibody dependent enhancement, a brief history and personal memoir. Rev Cubana Med Trop. 2002;54(3):171‐179. 15846943

[17] Sette A , Crotty S . Pre‐existing immunity to SARS‐CoV‐2: the knowns and unknowns. Nat Rev Immunol. 2020;20(8):457‐458. 32636479 10.1038/s41577-020-0389-z PMC7339790

[18] Cao WC , Liu W , Zhang PH , Zhang F , Richardus JH . Disappearance of antibodies to SARS‐associated coronavirus after recovery. N Engl J Med. 2007;357(11):1162‐1163. 17855683 10.1056/NEJMc070348

[19] Wu LP , Wang NC , Chang YH , et al. Duration of antibody responses after severe acute respiratory syndrome. Emerging Infect Dis. 2007;13(10):1562‐1564. 10.3201/eid1310.070576 PMC2851497 18258008

[20] Strömer A , Rose R , Grobe O , et al. Kinetics of nucleo‐ and spike protein‐specific immunoglobulin G and of virus‐neutralizing antibodies after SARS‐CoV‐2 infection. Microorganisms. 2020;8(10):1572. 10.3390/microorganisms8101572 PMC7650537 33066057

[21] Seow J , Graham C , Merrick B , et al. Longitudinal observation and decline of neutralizing antibody responses in the three months following SARS‐CoV‐2 infection in humans. Nat Microbiol. 2020;5(12):1598‐1607. 33106674 10.1038/s41564-020-00813-8 PMC7610833

[22] Cervia C , Nilsson J , Zurbuchen Y , et al. Systemic and mucosal antibody responses specific to SARS‐CoV‐2 during mild versus severe COVID‐19. J Allergy Clin Immunol. 2020;147:545‐557. 33221383 10.1016/j.jaci.2020.10.040 PMC7677074

[23] Iyer AS , Jones FK , Nodoushani A , et al. Dynamics and significance of the antibody response to SARS‐CoV‐2 infection. medRxiv. 2020. 10.1101/2020.07.18.20155374

[24] Gudbjartsson DF , Norddahl GL , Melsted P , et al. Humoral immune response to SARS‐CoV‐2 in Iceland. N Engl J Med. 2020;383(18):1724‐1734. 32871063 10.1056/NEJMoa2026116 PMC7494247

[25] Debisarun PAPS , Domínguez‐Andrés J , Moorlag SJCFM , et al. The effect of influenza vaccination on trained immunity: impact on COVID‐19. medRxiv. 10.1101/20201014202124982020

